# Organisational factors affecting emergency medical services’ performance in rural and urban areas of Saudi Arabia

**DOI:** 10.1186/s12913-021-06565-3

**Published:** 2021-06-07

**Authors:** Ahmed Ramdan M Alanazy, John Fraser, Stuart Wark

**Affiliations:** 1 School of Rural Medicine, Faculty of Medicine and Health, University of New England, Armidale, Australia; 2 King Saud bin Abdulaziz University for Health Sciences, Riyadh, Saudi Arabia

**Keywords:** Emergency Medical Service, Saudi Arabia, Rural, Urban, Paramedic

## Abstract

**Background:**

There is a disparity in outcomes between rural and urban emergency medical services (EMS) around the world. However, there is a scarcity of research that directly asks EMS staff in both rural and urban areas how service delivery could be improved. The aim of the present study is to gain insights from frontline workers regarding organisational factors that may underpin discrepancies between rural and urban EMS performance.

**Subject and methods:**

The study was undertaken in the Riyadh region of Saudi Arabia. Potential participants were currently employed by Saudi Red Crescent EMS as either a technician, paramedic or an EMS station manager, and had a minimum of five years experience with the EMS. Semi-structured interviews were undertaken between October 2019 and July 2020 with first respondents to a call for participants, and continued until data saturation was reached. All interviews were conducted in Arabic and transcribed verbatim. The Arabic transcript was shared with each participant, and they were asked to confirm their agreement with the transcription. The transcribed interviews were then translated into English; the English versions were shared with bi-lingual participants for validation, while independent certification of the translations were performed for data from participants not fluent in English. A thematic analysis methodological approach was used to examine the data.

**Results:**

The final sample involved 20 participants (10 rural, 10 urban) from Saudi Red Crescent EMS. Data analyses identified key organisational factors that resulted in barriers and impediments for EMS staff. Differences and similarities were observed between rural and urban respondents, with identified issues including response and transportation time, service coordination, reason for call-out, as well as human and physical resourcing.

**Conclusion:**

The findings identified key issues impacting on EMS performance across both rural and urban areas. In order to address these problems, three changes are recommended. These recommendations include a comprehensive review of rural EMS vehicles, with a particular focus on the age; incentives to improve the numbers of paramedics in rural areas and more localised specialist training opportunities for rurally-based personnel; and the implementation of national public education program focusing on the role of the EMS.

**Supplementary Information:**

The online version contains supplementary material available at 10.1186/s12913-021-06565-3.

## Background

In countries around the world, pre-hospital emergency medical services (EMS) provide essential on-scene care for individuals following an accident or the emergence / exacerbation of an acute or chronic health condition [1]. Without the timely intervention of an EMS, research indicates poor short- and long-term outcomes for individuals experiencing either a traumatic injury or serious illness [2, 3]. Improvements in pre-hospital care, including both treatment on-scene and during subsequent transportation to a medical facility, can result in considerable reductions in both mortality rates and the magnitude of ongoing health issues [4–6]. However, any attempts to achieve widespread improvements to EMS are complicated by a range of factors, including issues relating to navigating the local geography, coordinated response processes, access to and training in the use of life-saving and life-sustaining equipment, and the availability of both key staff and appropriate vehicles [7, 8]. While there is still only limited direct comparative research, there is evidence of disparity in outcomes between rural and urban EMS [9–11].

It is acknowledged that some of the generally accepted measures of EMS effectiveness, such as response time and transportation time interval, may be affected by simple geographic distance limitations in rural areas that cannot be easily overcome [12, 13]. Urban locations with well-established road networks and health infrastructure hubs are also not surprisingly associated with better pre-hospital EMS outcomes when compared to more isolated and remote rural areas [12]. Nonetheless, while it has been known for many years that living in a rural area in many developed countries will affect EMS outcomes e.g. [14], there is limited comparative research between urban and rural areas countries outside of the USA, UK and Australia [10]. Furthermore, there is a scarcity of research that has directly asked EMS staff in both rural and urban areas their perspectives on how EMS delivery could be improved.

This study is part of a wider project investigating the provision of EMS within the Riyadh region of the Kingdom of Saudi Arabia (KSA). Previous publications [12, 15, 16] have reported on quantitative data derived from retrospective analyses of Saudi Red Crescent EMS patient records. The knowledge gained from that stage of the project has informed this research, which aims to explore the reasons underpinning the identified EMS delivery differences between rural and urban areas, including response times, base equipment and staff training levels. The purpose of the present study is to gain insights from frontline workers, through personal interview, regarding any organisational factors in their day-to-day work practice that may underpin discrepancies between rural and urban EMS. This information will then be used to make recommendations to both improve the general operations of EMS in Saudi Arabia, and to also assist in closing the recognised service delivery gaps between rural and urban areas.

## Methods

Ethical approval for this project was granted by the University of New England’s Human Research Ethics Committee, the Saudi Arabia Ministry of Health Ethical Committee, The King Abdelaziz Medical Cities Ethical Committee, and the Saudi Red Crescent Authority. Every participant was given project information to review in advance, and they each provided individual consent prior to the commencement of interviews.

### Study setting

The study was undertaken in the region of Riyadh in Saudi Arabia. Makkah is the largest of the 13 administrative area in Saudi Arabia in terms of both population and EMS transportation cases and was the original location for the study. However, project advisors from the Saudi Red Crescent identified that the region was unlikely to produce data that could be representative for all Saudi Arabia. This was due to the fact that Makkah has significant annual religious events (pilgrimage) resulting in a substantial number of emergency medical events for temporary visitors and non-residents that the other regions do not experience. This was considered by the Saudi Red Crescent likely to result in data that did not represent Saudi Arabia as a whole. As such, Riyadh region, which has the second-highest population and number of transported cases, was selected as the setting for the project. Riyadh region has an estimated population of eight million people in a geographic area of 400,000 km². Located in Riyadh region is the capital city Saudi Arabia, which is also called Riyadh.

Servicing the residents of Riyadh region is the Saudi Red Crescent EMS, which is the primary EMS for all civilians across Saudi Arabia; hospitals use a separate Ministry of Health ambulance system for transportation of patients [1]. Riyadh region has its own Dispatch and Call Centre in Riyadh city, which is managed by Saudi Red Crescent Authority and covers both rural and urban locations in the region. The Saudi Red Crescent EMS current approach is based on the Anglo-American model that transports emergency patients to the Saudi Arabian health system of community-based hospital emergency departments, while being supported clinically trained staff including paramedics and emergency technicians. Saudi Red Crescent has approximately 400 ambulance stations across Saudi Arabia [1].

### Participants

The participants for the study were drawn from a cohort of Saudi Red Crescent EMS staff living in Riyadh region and who had completed previous components of the larger study. At that time, they were asked to indicate their willingness to be subsequently interviewed. An expression of interest was sent to all of the individuals who had consented to the follow-up interview, and they were asked to respond if they were still willing to be interviewed. All potential participants were advised at this time that their names would not be used in any reporting of the data, and that their anonymity would be ensured as best possible.

To be considered eligible for interview, possible participants had to be currently employed by Saudi Red Crescent as either a technician (EMT), paramedic or a EMS station manager, and have a minimum of five years experience with the EMS. It was known in advance that it was highly likely that all participants would be male, as this is reflective of the demographic makeup of the Saudi Red Crescent EMS workforce [17, 18]. In accordance with the geographic classification provided by the Saudi Red Crescent, individuals working in Riyadh City were classified as ‘urban,’ while participants residing in all other areas of Riyadh region were defined as ‘rural.’

### Study design and materials

The study used a hermeneutic phenomenology research methodology [19] to guide both its development, implementation and analyses. This approach was deliberately chosen as the most appropriate mechanism to gain a nuanced understanding of EMS delivery in rural and urban areas of Saudi Arabia. The focus of this study was on the lived experience of frontline staff, with the hermeneutic phenomenology methodology underpinning the reflective analyses of the qualitative dataset [20]. Semi-structured individual interview was the preferred method for collection of data from both rural and urban participants [20]. In order to ensure the sample included appropriate representation from both rural and urban EMS staff, a pre-determined figure of 8–12 interviews for each category (rural or urban worker) was nominated [21, 22]. Interviewees were determined on the basis of first-respondents, and the interviews continued until data saturation [21] was reached.

Prior to commencement of the face-to-face interviews, an interview guide was developed and piloted. The initial questions within the guide were established through review and discussion amongst the team of the quantitative data gained in previous stages of the project [12, 15, 16]. This draft guide was trialled with two Saudi Arabian EMS workers, with each of these participants asked to provide feedback on the wording of the questions, and to note any other issues or concerns. Their feedback was reviewed by the research team, and some minor changes to the wording of questions was made to ensure clarity for participants. This revised interview guide then became the template document for the semi-structured interviews with EMS workers, and is included as a supplementary file.

### Data collection procedure

There were two stages to the data collection which occurred over a period of 10 months between October 2019 and July 2020. Stage one of the data collection involved face-to-face interviews (by the lead author, an experienced male Saudi paramedic) with participants in Saudi Arabia. Each participant was interviewed by themselves in a quiet room at their home EMS base in Riyadh region. All interviews were conducted in Arabic, and commenced with the interviewer introducing himself and a general discussion around the purposes of the study before continuing onto the questions in the interview guide. No participants decided to withdraw from the study, either during this initial introduction stage or following completion of the interview. Field notes were taken by the lead author following each interview.

The interviews each lasted between approximately 30 min and one hour, and the audio recordings of these interviews were transcribed verbatim. To assist with validation and trustworthiness of the data [21], a process of confirmation of content was undertaken with the participants following initial transcription. The Arabic transcripts were shared with the participants, and they were asked to confirm their agreement with the transcription. This process did not identify any areas of concern in regard to the transcription. The transcribed interviews were then translated into English by the lead author. Many of the participants were fluent in both the Arabic and English languages; the English versions were therefore also shared with bi-lingual participants for validation of the translation. For the data from participants who were not fluent in English, independent certification of the translation was performed. No issues were identified with the transcription and translation process by participants or during certification.

Initial analyses of the data (see detailed description of analysis below) by the entire team could not begin until transcription and translation into English had occurred. When this occurred, it became evident that there were emerging issues around gender that had not necessarily been raised with all participants. As such, it was determined that there was a need for a second round of interviews with the same participants to further explore these concepts. This resulted in another stage of data gathering, and which involved the use of an online technology (Zoom) to conduct follow-up interviews. The use of Zoom was necessitated by restrictions associated with the Covid-19 pandemic, and an ethics variation was approved to accommodate this change in approach. The process for data transcription and translation was the same for the second round of interviews as previously described for the initial face-to-face interviews.

### Analysis

The dataset was developed from interviews with a total of 20 participants, with an even split of 10 rural and 10 urban EMS personnel. Data saturation was considered to be reached after nine rural and eight urban interviews, however it was agreed to complete ten interviews in each location as they had been pre-arranged with participants. No new issues emerged in these final three interviews, confirming the decision regarding data saturation.

Prior to commencement of analyses, an anonymous identifier code was assigned to each participant. This code also included their location as being either rural or urban, and their total years of experience. A gender identifier was ultimately not required as all participants were male.

In order to undertake analysis of the data, the research team adopted a thematic analysis methodological approach using the framework of Braun & Clarke [22]. Initially, each of the three members of the research team independently read the English translations of the interview transcripts. The purpose of this first review was generate preliminary concepts and to establish a general idea of any key themes that were emerging from the data. After all members had completed this stage, a team meeting was held in which agreement was reached on an initial coding structure. Each researcher then used the coding structure to independently code the statements within each of the transcripts. This process also involved each member of the team considering and documenting the perceived context of the statements for each participant.

Two members of the research team then met and jointly reviewed all of the transcripts after initial coding, discussed the emerging themes, and then agreed on a final coding structure. The third member of the research team had been nominated in advance to act as arbiter in the event of any disagreements that could not be resolved, but this was not required. Following confirmation of the final structure, the transcripts were coded by the lead researcher and then refined into thematic areas by the entire team.

## Results

The sample was composed of 10 EMTs (6 rural and 4 urban), 3 paramedics (0 rural and 3 urban) and 7 managers (4 rural and 3 urban).The basic demographic data for the two samples [rural and urban] was similar. The mean age of the rural participants was 35.25, which was marginally higher than the mean age of urban participants at 32.5. In light of this slight chronological mean age difference, it is not surprising that the rural participants also stated that they had more experience working for the EMS. However, the difference again was small, with rural participants reporting a mean 11.5 years of experience compared to urban EMS staff who reported an average 9.9 years of experience.

Initial analyses of the entire dataset resulted in the identification of three separate themes; Organisational Factors; EMS Personnel Issues; and Patient Factors. The focus of this paper is on the theme of Organisational Factors, with EMS Personnel Issues and Patient Factors being subject of a separate publication. Further analyses of the theme of Organisational Factors identified a number of sub-themes, including response and transportation time, service coordination, reason for call-out, as well as human and physical resourcing. These sub-themes are described in the following section under the general headings of *Differences between rural and urban areas* and *Similarities between rural and urban areas*. Select quotes that provide additional understanding of the sub-themes have been included. To provide context for the reader, all quotes are linked to the relevant participant’ identifier (job role, location and years of experience).

### Organisational factors – differences between rural and urban areas

Participants were asked about potential organisational differences between urban and rural areas in relation to the performances of EMS. One of the first issues identified by staff in both rural and urban areas was response time. In the rural areas, it was a common for the EMS to take a longer time in reaching the scene of an emergency when compared to the cases in urban locations. This longer response time was then linked to incidences of patients being at risk of worsened medical conditions. One rural EMT (8 years experience) said:

“*I think the difference is that in the rural area the ambulance stations are far away from each other so we need to travel longer distances. One time our crew had to travel 40 kilometres to reach the scene and when we arrived, we found out that 3 cases were involved in that accident so we had to wait for another 40 minutes to get the backup”.*

Previous research by the authors [15] identified differences between rural and urban areas in terms of response times across the day, with morning’ response times being lower when compared to afternoon and night in urban areas, while afternoon’ response times were lower than night and morning in rural locations. When asked their thoughts about this disparity, the participants suggested that ‘rush hour traffic’, particularly in the afternoon and evening, was a factor that lead to the observed urban pattern. An urban EMS Manager (15 years experience) noted: “*Sometimes we need more time in the rush hour for example, we can reach a location in 4 mins at 3 AM but we need more than 20 mins in the rush hours due to traffic*”. Other than having more EMS stations, there were no suggestions for organisational change that could improve this issue. However, it was noted that a lack of equipment and limited guidance from central dispatchers (see below for further discussion of these issues) may inhibit the ability of rural EMS to find locations, and particularly at night, leading to the longer response time.

Transportation or conveyance of patients to medical facilities for further medical attention in rural areas was also identified to be a lengthy process because of the limited number of hospitals that are often a considerable distance from the location of the accident or medical emergency. A rural EMT (8 years experience) identified the following concerns:

*“We take longer time in the rural area. Sometimes we need to travel for 40 minutes and we take the 10 to 15 minutes in the scene. Then we need to travel even further to the hospital which is 40 minutes away from us or even more, so in total we take almost 2 hours”*

It was accepted that the geographic distance factor may be difficult to overcome, again without significant infrastructure development, but the equipment required to reach patient location in rural areas also was noted as having an effect on duration and response time. One rural EMT (6 years experience) identified that: *“In rural areas, sometimes we are dispatched to a long distance location. Some locations need 4 × 4 cars and some locations are in the middle of nowhere.”* He further recognised the need for dispatch services to have a solid understanding of the geographic area, otherwise time is wasted in simply finding the appropriate location: “*We need a dispatcher with good knowledge of each area. For example, for the area of West Kharj we need a dispatcher from that area who knows the area very well. Apart from that, we need a GPS system*.”

There were reported differences from the participants in relation to reasons for an emergency call. In particular, trauma cases were reported as being more common in rural areas: “*Most of our cases in the rural areas are trauma while the urban cases are medical”* (rural EMT, 9 years experience). In turn, this has implications for how services are provided, and what essential equipment and medications are required in the rural EMS stations and hospitals. Rural interviewees in particular believed that there was a clear discrepancy in the distribution of medical resources, both human and physical, between the urban area and rural area, and that the rural locations are marginalised when it comes to distribution of resources. A rural EMS Manager (15 years experience) said *“We need more attention from our employer as we need new buildings for ambulance stations. We also need new cars as our cars are slightly older compared to those in the urban areas.”*

It was also recognised that there is a higher proportion of EMTs, rather than the higher trained paramedics, in rural areas than in urban areas. This is problematic, as only paramedics can administer medication or perform certain procedures on-scene. A rural EMT (17 years experience), who had also worked in urban areas, noted that:

*“I think it is really important that we have paramedics in the rural area because we sometimes handle critical cases. For example, if we face pneumothorax, as an EMT our scope of practice will not allow us to do needle decompression, only a paramedic can do it yet it is a life saving procedure. So if we have a paramedic we could save the patient’s life.”*

Another rural EMT (11 years experience) confirmed that additional paramedics are needed in rural areas: “*Yes indeed. This is because the paramedics can give medications that will help in improving the cases.”* When asked a follow-up question for ideas on improving the number of paramedics in rural areas, this participant felt that incentives such as scholarships could assist. They suggested that: *“I think we need more scholarship opportunities because it is really costly to study paramedical science. With the scholarship, most of the people would study it.”* An urban station manager also agreed with this suggestion for rural areas, commenting that: *“We need to get the chance from our employer to get a scholarship to study as paramedics.”*

### Organisational factors – similarities between rural and urban areas

Not all of the organisational issues reported by the participants were perceived to necessarily be different due to the geographic location. Both rural and urban interviewees indicated how factors, including disaster planning liaison with other agencies, EMS autonomy, coordination of medical equipment, and the type of EMS model in use, had a considerable impact on their ability to perform at the level they would desire. These operational processes were seen to result in unnecessary delay, and therefore patients were slower to receive emergency medical assistance.

A specific concern raised was liaison with other agencies. It was noted that there is poor coordination between EMS services and the other support agencies. One rural EMT (9 years experience) expressed his frustration in this area: “*the lack of coordination between the ambulance and the other organisations as sometimes the police and fire fighters backup take a longer time to arrive at the scene.”* There was also a need for the careful coordination of medical services. One interviewed EMT (urban,11 years experience) noted that *“We need more coordination with the ministry of health ambulances because each hospital has their own ambulances so when their chronic patients need to be taken to their hospital they can transport him/her.”* This issue reflected the concerns regarding coordination between the Saudi civilian and health ambulance models, and avoiding unnecessary duplication of services.

The participants were also asked their thoughts on the high numbers of non-transported cases following callout across both rural and urban areas [16]. This issue was recognised by participants as being of concern, as the limited EMS resources are potentially diverted away from critical care cases to non-urgent callouts. One of the key issues identified was a perceived lack of knowledge and general awareness of the wider public regarding the roles and responsibilities of the Red Crescent EMS. As such, an outcome of non-transportation was seen to be often the result of calling for emergency assistance for minor health problems. When asked for solutions to this problem, a rural EMT (9 years experience) stated that: “*The public need to have an awareness about the role of the EMS - I think public awareness is really important. We need to teach people when they can call for the ambulance and not calling just for a minor injury in the finger for example.”*

## Discussion

This study reports on interviews conducted with 20 Red Crescent EMS personnel from across rural and urban areas of Riyadh region in Saudi Arabia. The goal was to gain a more nuanced understanding of the organisational factors that result in previously identified service delivery issues in this region [12, 15, 16]. Through thematic analysis of these interviews, it was possible to identify factors that contribute towards the existing disparities between rural and urban regions, and also common areas of concern for EMS personnel. These are discussed below under the three themes of Response and transportation time; Composition of the workforce; and Coordination of support for frontline staff.

### Response and transportation time

The response time interval is a critical factor noted as contributing to poor EMS outcomes [10]. However, previous research around the world has identified that attempts to remediate problems with response times in rural areas are influenced by numerous factors including local geography, staff training, and the availability of both key personnel and appropriate equipment [7, 8, 12, 13]. Urban locations which feature well-resourced infrastructure are not surprisingly associated with better pre-hospital EMS outcomes [12]. Specifically in the rural settings in the current study, EMS staff recognized that they took a longer period of time to reach patients, and to then transport them from the scene following initial treatment. If the patient had been involved in a traumatic accident or suffered a serious medical emergency, a longer response time could have significant implications and result in the need for medical interventions that may have been otherwise avoided. Managing to reduce the response time could significantly reduce the risk prior to admission of a patient to a healthcare facility.

It is acknowledged that the simple geographic distance factor in rural areas of Saudi Arabia will always limit the capacity of any EMS to reduce response time, and also the subsequent transportation time [15]. However, there was a perception amongst participants that rural EMS had older vehicles that were perhaps less reliable than those in urban areas, and that the need for specialist vehicles, such as four-wheel drive enabled options for difficult terrain, were not also available in all rural locations. A comprehensive review in rural areas may identify where upgrading of older vehicles may result in an improvement in response time, and therefore lead to better patient outcomes.

The on-board availability of Global Positioning Systems (GPS) seemed to vary across locations, and is not believed to be solely a rural issue. Instead, it was considered that a lack of GPS was possibly reflective of the age of the vehicle. However, as noted in the Results, it was recognised that rural areas often had older vehicles and that this may have contributed to the problem appearing worse in rural locations. Ensuring that all vehicles had GPS, and also that all staff were trained in using on-board GPS, may assist in finding remote locations and thus reducing the response time.

Another issue that was considered to underpin delays in response time was the number of callouts that subsequently resulted in non-transportation [16]. It was not possible to establish from this data whether there was a difference between rural and urban areas in this regard, and public education may assist to at least partially overcome this problem. However, it is also hypothesised that people living in more remote rural locations that have limited public transport options may be more likely to call the EMS for non-critical assistance. This could include calls for both minor medical conditions or to simply access the nearest hospital for routine health checks. As such, a wider consideration of access to health-care support in rural areas may also be appropriate.

### Composition of the workforce

A national study of EMS professionals in Saudi Arabia found that there were considerably more frontline staff with Diploma and lower qualifications than those with Bachelor, Masters or PhD level degrees [18]. However, the participants in the current study believed that there was further gradation to this issue, and noted additional discrepancy between urban and rural areas in this area. They perceived that the EMS personnel in rural areas often lacked the same base level of training as their urban counterparts to support traumatic injuries, with fewer paramedics and therefore a greater proportion of EMTs who have a lower level of education. This skills gap is magnified by the higher likelihood of trauma in rural areas of Saudi Arabia, potentially arising from high speed accidents and industrial mishaps [12]. Further compounding this disparity is the fact that the resourcing for on-site care in such situations is also limited in rural locations [9], leading to higher mortality rates when compared to urban centres. Prior research [12] indicates a number of issues underpinning this training gap in Saudi Arabia, and previous recommendations for localised training and incentives for rural paramedics are again suggested as possible solutions to address this workforce imbalance.

### Coordination of support for frontline staff

The coordination and information flow during dispatch of EMS services was reported as causing problems in responding quickly and appropriately to emergency situations. This appeared to be more problematic in rural areas, with centralised dispatch services in larger urban locations naturally lacking a comprehensive knowledge of rural areas. This was perceived to affect timely access to the patient as a consequence of limited directions for the responding EMS. The problem is further complicated by the previously noted issue of some rural EMS vehicles lacking GPS to support them reaching a patient’s location.

In general, this is a complex issue as a centralised dispatch model does have a number of positive aspects, including consistency of response in addition to general operational efficiencies and cost-savings [23, 24]. Equally, it is not feasible to expect each dispatch officer to have extensive knowledge of all areas of both rural and urban Saudi Arabia. Ensuing that all vehicles have reliable GPS could mitigate some of these issues, however it is also suggested that it may be useful for all dispatch officers to spend some time on an annual basis in rural areas to ensure that they have at least a general understanding of the local environments and potential hazards that may impede accurate identification of rural locations [25].

### Limitations

This project was designed and undertaken with a specific focus on one geographic region, Riyadh, in the Kingdom of Saudi Arabia. While an attempt was made to choose a location that may be considered relatively representative of Saudi Arabia, it is acknowledged that the reporting of personal experiences and systematic problems may vary considerably on location, and depending on what resources are available locally. As such, any comparisons to other settings, either within the Kingdom or across other similar countries, should be carefully considered in light of the local settings and also variations in legislation regarding the provision of emergency medical services.

## Conclusions

The data arising from this project allowed for the identification of key issues impacting on EMS performance across both rural and urban areas of Riyadh region in Saudi Arabia. In particular, there were a number of problems cited by the participants that were specific to rural locations and which resulted in disparities in service delivery across the region. These issues included the lack of vital equipment, lower levels of base training and qualifications, public uncertainty about the role of the EMS, and poor service coordination of frontline staff.

In order to address these problems, three possible changes to either policy or practice that are not reliant on extensive overhauling of the existing systems are recommended. These are:

1. While it is acknowledged that the larger geographic distances in rural areas will naturally result in longer response time, this can be potentially reduced by ensuring that all vehicles are appropriate for the environment, and equipped with the necessary equipment, including GPS. It is recommended that a comprehensive review of all rural vehicles is undertaken to identify any gaps, with a particular focus on the age of the vehicle.
2. It is recommended that more localised specialist training opportunities are provided for rurally-based EMS personnel as part of an upskilling process to address the disparity between the rural and urban workforces. Further, it is suggested that incentives, and specifically scholarships for rural residents to study paramedicine at university, be considered to address this imbalance.
3. A national public education program, focusing on the role of the EMS and what staff can and cannot do, would be worthwhile in addressing some of the perceived misunderstandings regarding the role of the EMS by the general public.

## Supplementary information

**Additional file 1.**

## Data Availability

De-identified data is available on reasonable request from the lead author.

## Abbreviations

EMS: Emergency Medical Services
EMT: Emergency Medical Technician
GPS: Global Positioning System
KSA: Kingdom of Saudi Arabia
