# Supplementary material for: Organisational factors affecting emergency medical services’ performance in rural and urban areas of Saudi Arabia
Source: BMC Health Serv Res. 2021 Jun 7;21:562. doi: 10.1186/s12913-021-06565-3 (PMC8183589; doi:10.1186/s12913-021-06565-3)
Supplement: Supplementary file 1 — Additional file 1. [file 12913_2021_6565_MOESM1_ESM.docx]

***Semi-structured Interview Guide (Phase 1)***

**Commencing Demographic Questions**

Age:

Gender:

Years of experience in EMS in Saudi Arabia:

Current work location: Rural Urban Both

Previous work locations: Rural Urban Both

Qualifications:

**Questions about Operational Issues**

***1- Can you outline your experiences to date working in the EMS system in Saudi Arabia?***

Prompts:

What are the most rewarding aspects of your role?

What aspects of your role detract from your work satisfaction?

***2- In general, what do you think are the strengths of the current model of care provided by EMS for patients?***

***3- In general, what do you think are the weaknesses of the current model of care provided by EMS for patients?***

***4- In your opinion, does the provision of EMS services in rural areas differ from those in urban areas?***

Prompts:

Do you think the severity or complexity of the cases is the same?

Are the available resources similar?

Are there any specific obstacles in a location that may inhibit service delivery?

***5- Do you think there is a significant difference in the response time between rural and urban emergency medical services?***

Prompts:

if YES: What things do you think may contribute to this difference?

If NO: Our analysis of Red Crescent data showed that there was a significant difference. What things do you think may have contributed to this difference?

***6- Can you make any suggestions or recommendations on how to improve the response times in either rural or urban areas, or both?***

***7- Do you think there is a difference in duration time between rural and urban emergency medical services?***

Prompts:

if YES: What things do you think may contribute to this difference?

If NO: Our analysis of Red Crescent data showed that there was a significant difference. What things do you think may contribute to this difference?

***8- Do you think that the location of the patient will have an effect on the response and duration time between urban and rural areas?***

Prompts:

if YES: What things do you think may contribute to this difference?

If NO: Our analysis of Red Crescent data showed that it took a significantly longer time to transport a patient from home when compared to a street or highway location. What things do you think may contribute to this difference?

***9- The number of female patients who are transported to hospital is far lower than the number of male patients in both rural and urban areas. What do you think are the reasons for this difference?***

Prompts:

Are there any specific cultural issues that may cause this?

Do you think having female paramedics may see an increase in the number of female patients using EMS?

***10- Our analysis of Red Crescent data showed that rural areas had a much higher rate of head injury than the urban area. Can you think of any reasons for this finding?***

***11- Can you make any suggestions or recommendations for any changes to the job role of paramedics/ non paramedics?***

Prompts:

Can you comment on availability of equipment, support, training and/or education of paramedics/ non paramedics that may improve patient outcomes?

Do these recommendations apply to rural or urban areas, or both?

**Patient Outcomes**

***1- In general, can you make any suggestions or recommendations for improving outcomes for patients who use the EMS in Saudi Arabia?***

***2- Our analysis of Red Crescent data showed a large number of patients in both urban and rural areas refused transportation. Can you think of any reasons for the large number of reported non-transported cases?***

***3- The urban areas reported a higher non-transported rate than rural areas. Can you think of any reasons for this?***

***4- Do you think there would be a difference in the length of stay in hospital between rural and urban cases?***

Prompts:

Yes-Why do you think this would be the case?

No - Our analysis of Red Crescent data showed that there was a significant difference in the length of stay in hospital between rural and urban areas. Why do you think this would be the case?

***5- Do you think there would be a difference in the length of stay in the Intensive Care Unit between rural and urban cases?***

Prompts:

Yes -Why do you think this would be the case?

No - Our analysis of Red Crescent data showed that there was a significant difference in length of stay in ICU between rural and urban areas. Why do you think this would be the case?

***6- Do you have any further questions or comments that you would like to make in relation to the delivery of EMS in Saudi Arabia?***

**Thank you for your time.**

***Semi-structured Interview Guide (Phase 2)***

**Follow-up interview questions (Zoom )**

1. ***A number of paramedics mentioned that they do not have the authority to refuse transportation from the scene if the patient requests it. Can you confirm whether this is your understanding of your authority?***

If not, what is your understanding of your ability to make on-scene decisions?

1. ***If you were able to make on-scene decisions regarding non-transportation, do you believe that you have sufficient medical training and knowledge to appropriately make such decisions?***
2. ***I would like to know more about how existing EMS staff would feel about working with more female EMS staff. Can you tell me your thoughts around this?***
3. ***How do you think female EMS staff would be accommodated into the service?***

Prompt: Would it be a mixed EMS crew?

1. ***What difference, if any, do you think would happen if EMS were allowed to take a female nurse along to sites to support female patients?***

**Thank You**
